# Preformulation Studies on Solid Self-Emulsifying Systems in Powder Form Containing Magnesium Aluminometasilicate as Porous Carrier

**DOI:** 10.1208/s12249-014-0247-z

**Published:** 2014-12-11

**Authors:** Anna Krupa, Jakub Szlęk, Benedykt R. Jany, Renata Jachowicz

**Affiliations:** 1 Department of Pharmaceutical Technology and Biopharmaceutics, Faculty of Pharmacy, Jagiellonian University, Medical College, 9 Medyczna Str, 30-688 Cracow, Poland; 2 Marian Smoluchowski Institute of Physics, Jagiellonian University, Cracow, Poland

**Keywords:** dissolution, ibuprofen, labrasol, magnesium aluminometasilicate, self-emulsifying powder

## Abstract

The influence of alkaline and the neutral grade of magnesium aluminometasilicate as a porous solid carrier for the liquid self-emulsifying formulation with ibuprofen is investigated. Ibuprofen is dissolved in Labrasol, then this solution is adsorbed on the silicates. The drug to the silicate ratio is 1:2, 1:4, and 1:6, respectively. The properties of formulations obtained are analyzed, using morphological, porosity, crystallinity, and dissolution studies. Three solid self-emulsifying (S-SE) formulations containing Neusilin SG2 and six consisting of Neusilin US2 are in the form of powder without agglomerates. The nitrogen adsorption method shows that the solid carriers are mesoporous but they differ in a specific surface area, pore area, and the volume of pores. The adsorption of liquid SE formulation on solid silicate particles results in a decrease in their porosity. If the neutral grade of magnesium aluminometasilicate is used, the smallest pores, below 10 nm, are completely filled with liquid formulation, but there is still a certain number of pores of 40–100 nm. Dissolution studies of liquid SEDDS carried out in pH = 1.2 show that Labrasol improves the dissolution of ibuprofen as compared to the pure drug. Ibuprofen dissolution from liquid SE formulations examined in pH of 7.2 is immediate. The adsorption of the liquid onto the particles of the silicate causes a decrease in the amount of the drug released. Finally, more ibuprofen is dissolved from S-SE that consist of the neutral grade of magnesium aluminometasilicate than from the formulations containing the alkaline silicate.

## INTRODUCTION

In the recent years, the adsorption of lipid drug formulations onto porous solid carriers has been the aim of several studies (1–16). It is known that in the case of insoluble drugs, an ideal solid carrier should have a high loading capacity, no toxicity, no negative impact on the drug stability, and assure complete drug release in the gastrointestinal tract. The process of liquid adsorption on mesoporous solid carriers should also be easy to scale-up.

The functionality of pharmaceutical mesoporous silicates, such as silicon dioxide, calcium silicate, or magnesium aluminometasilicate in the preparation of solid self-emulsifying (S-SE) drug delivery systems with lansoprazole or gentamycin sulfate of improved bioavailability have been reported (9,10).

With regard to ibuprofen, a controlled drug delivery has been obtained by soaking the synthetic silicates, such as TUD-1, MCM-41, or SBA-15 in hexane and ethanol drug solution or by co-spray drying (13–16). Moreover, the modification of the synthetic silicate surface by amino functional or another alkaline group was also found suitable to control the release rate of acidic drugs (16). The pore size and specific surface area of the solid carrier determined both the drug release rate and the silicate loading capacity (6,9–11,15,16).

The previous studies on the physical mixtures, composed of ibuprofen and the neutral grade of magnesium aluminometasilicate, showed that the silicate protected ibuprofen from evaporation at high temperatures (17). It was due to the formation of a salt in the reaction between the carboxyl group of ibuprofen and the hydroxyl groups from the surface of Neusilin US2. The reaction was initiated by blending the drug and the excipient at room temperature. Similarly, the beneficial effect of magnesium aluminometasilicates on the stability of amorphous quinapril hydrochloride after storage at high humidity has been reported (18). Several studies have also been carried out on the amorphization of crystalline drugs by co-grinding with silicates (19).

In general, liquid lipid formulations destined to be adsorbed on solid carriers are multicomponent and they are composed of an oil phase, surfactant, and co-surfactant (1,2). The presence of many liquid excipients in solidified formulations may cause problems during compression, capsule filling, or granulation (2–4). Moreover, due to the high viscosity of the lipid formulation, the application of organic solvents may be necessary for effective impregnation of the carrier (15,16). Thus, only a few reports deal with the analysis of S-SE formulations composed of one liquid excipient, in which an insoluble active ingredient (API) is dissolved or dispersed (9,10).

Several reports focus on the functionality of Labrasol to improve the dissolution and bioavailability of poorly water soluble drugs (2,9–11). Labrasol forms micelles in water, which results in improved solubility of the API. The fluidity of the gastric and intestinal mucosa increases, and the drug bioavailability is higher (9). It has been shown that Labrasol improves the solubility of ibuprofen from 0.02 mg/mL to almost 150 mg/mL (20).

Furthermore, there are no scientific reports on the influence of magnesium aluminometasilicates as carriers that differ in pH on the preparation of the solid SE powder with ibuprofen. In the present study, the lipid formulation to be adsorbed on the silicate surface is composed of only one liquid excipient. Labrasol as a non-toxic, non-ionic surfactant of self-emulsifying properties is used as a solvent for ibuprofen. It consists of a mixture of PEG-8 caprylic/capric glycerides that has wetting and solubilizing properties.

Therefore, the aim of the present study is to prepare S-SE formulation with ibuprofen in the powder form by the adsorption of the drug solution in Labrasol onto particles of either the neutral or alkaline grade of magnesium aluminometasilicate. Attention is paid to using a minimal number of excipients during the manufacturing process. Since the solubility of ibuprofen depends on pH, the influence of carrier properties on the drug dissolution mechanism is investigated. S-SE systems are prepared by simple blending of the drug solution with heated silicate powder. There are no organic solvents used for the carrier impregnation. The loading capacity of silicates as well as the influence of the silicate grade and Labrasol content on the possibility to obtain S-SE formulation in powder form is analyzed.

## MATERIAL AND METHODS

Ibuprofen (IBU) was purchased from Shasun Chemicals and Drugs, India. Labrasol was supported by Gattefosse, France. Two grades of magnesium aluminometasilicate, *i.e.*, Neusilin US2 and Neusilin SG2, were kindly donated by Fuji Chemical Industry, Japan. Hydrochloric acid 37% was obtained from Merck Millipore, Poland. Potassium dihydrogen phosphate and sodium hydroxide of analytical grade were purchased from Avantor Performance Materials Poland S.A.

### Preparation of SE Formulations in Powder Form

Nine SE formulations containing either Neusilin SG2 (S1–S9) or Neusilin US2 (U1–U9) as a solid carrier were prepared (Table I). Labrasol was heated at 60°C, using water bath (LW-2, Poland) for 5 min. Its amount was 150, 300, or 600 μL, respectively. Then, 50 mg of ibuprofen was added and the mixture was heated under stirring for 2 min until the drug was dissolved. Magnesium aluminometasilicate was added gradually to this solution. The amount of the carrier was 100, 200, or 300 mg. Stirring continued until the mixture was uniform. After cooling, the formulations were stored at the ambient temperature for 24 h, then they were passed through a 500-μm mesh sieve.

**Table I:**
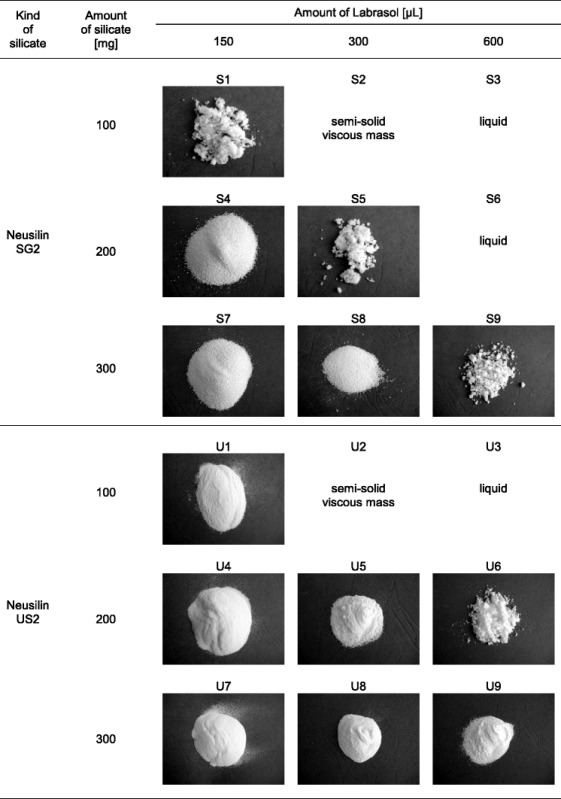
Consistency of S-SE With Ibuprofen. Images Taken by CCD Camera

Six physical mixtures containing ibuprofen and Neusilin SG2 (MS1–MS3) or Neusilin US2 (MU1–MU3) in 1:2, 1:4, and 1:6 ratio were also prepared as a reference. The drug was dry-blended with the silicate in a mortar for 2 min.

### Morphological Studies

The morphology of ibuprofen and both grades of magnesium aluminometasilicates were analyzed using the scanning electron microscope (SEM) Hitachi S-4700 (Japan). The powder was adhered to the sample holder by a double-sided copper tape. Its surface was coated with carbon using 208 HR carbon sputter coater (Cressington, USA). Images were taken at ×400 and ×1000 magnification.

In order to analyze the texture of the sample surface, SEM FEI Quanta 3D FEG (USA) in low vacuum mode (80 Pa water) with electron energy 30 keV was used. Images were taken at ×1000 and ×250,000 magnification.

The morphology of the samples prepared was also analyzed, using the CCD camera (DSC-W50, Sony, China).

### Texture Analysis

A specific surface area, pore surface area, volume, and pore size distribution of magnesium aluminometasilicates as well as S-SE samples were determined by the nitrogen adsorption/desorption method. The experiments were performed at 77 K, using the volumetric analyzer ASAP 2010 (Micromeritics, USA).

Prior to the adsorption, the samples were degassed at 313 K for 26 h in vacuum conditions. A specific surface area was determined from the adsorption isotherms by applying the Brumauer-Emmett-Teller (BET) and Langmuir equations in the relative range of pressure 0.05 < p/p_0_ < 0.25, taking the cross-section area of the nitrogen molecule to be 16.2 Å^2^ (21).

The mesopore surface area, the mean mesopore volume, and the mesopore size distribution in the range from 1.7 to 300 nm were determined, using the Barret-Joyner-Halenda (BJH) method and ASAP 2010 v.3.02 software (22). In the BJH method, it is assumed that the physical adsorption of nitrogen on the pore walls at the liquid nitrogen temperature and the capillary condensation are mechanisms that determine an equilibrium between the gas phase and the adsorbed phase during nitrogen desorption. There is a relationship between thickness of physically adsorbed layer and relative pressure. Therefore, nitrogen desorption isotherms can be used to calculate the distribution of mesopore volume and area with respect to pore radius (22).

The micropore surface area and pore volume were calculated with t-plot method. In this method, the experimental test isotherm is redrawn as a t-curve, in which the volume of gas adsorbed is plotted *versus* the standard multilayer thickness (t) of the reference non-porous material at the corresponded p/p_0._ The differences between the shape of the experimental isotherm and the standard isotherm give the information about the micropore volume and micropore surface area (23).

### X-ray Powder Diffraction

The samples of ibuprofen, silicates as well as formulations prepared were analyzed by X-ray powder diffraction (XRPD). The measurements were performed using Philips X’Pert APD diffractometer. The samples were exposed to X-ray radiation (Cu-Kα) with the wavelength of 2 Å and a 0.02-mm Ni-filter. The X-Ray tube was set up at a voltage of 45 kV and a current of 40 mA. The rate of the scanning was 0.02°/min at a range of 2–60 2Θ.

### Dissolution Studies and Kinetic Analysis

Ibuprofen release from SE powder was examined in the automated pharmacopoeial paddle dissolution apparatus Hanson Research Dissolution Station SR8 Plus with the autosampling device Dissoette II (USA). A sample of liquid SE formulation or powder containing 10 mg of ibuprofen was used for the study to assure sink conditions. The dissolution test was performed in 1000 mL of HCl 0.1 mol/L or phosphate buffer of pH = 7.2 prepared according to Ph. Eur. 8th edition at 37°C ± 0.5°C. The paddle rotation speed was set at 50 rpm. The samples of 5 mL (*n* = 3) were withdrawn through the polyethylene filter (10 μm). The amount of ibuprofen dissolved was determined spectrophotometrically, using UV–VIS spectrophotometer Jasco VT-530 (Japan) at 223 nm wavelength. Standard deviation (SD) and relative standard deviation (RSD) were calculated.

KinetDS 3.0 free license software was used to determine the kinetics of ibuprofen dissolution (24). The dissolution curves were fitted to the zero order, first order, Korsmeyer-Peppas, Michaelis-Menten, Hixson-Crowell, and Higuchi mathematical models (24,25). The goodness of fit was evaluated using one-way ANOVA test to determine if the differences between the coefficients of determination (*r* ^2^) are statistically significant (*P* < 0.05) as well as by chi-square test (*χ* ^2^) with established critical chi-square value of 0.7102 (lower-tail) for *α* = 0.05 and degrees of freedom (df) equal to 4. The null hypothesis was tested if there were no statistically significant differences between models’ fitted curves and observed dissolution points. Moreover, to select the best model within the formulation, Akaike information criteria (AIC) parameter was calculated by KinetDS3.0 software (24).

The model-independent parameters, such as dissolution efficiency (DE) and mean dissolution time (MDT) were also calculated by KinetDS 3.0 software according to Eqs. 1 and 2, respectively (24).

1 $$ \mathrm{D}\mathrm{E} = \frac{{\displaystyle {\int}_0^t}Q\mathrm{d}t}{Q \max \times t}\times 100 $$

2 $$ \mathrm{M}\mathrm{D}\mathrm{T}=\frac{{\displaystyle {\sum}_{j=1}^n}t{}_j{}^{AV}\times \varDelta Qj}{{\displaystyle {\sum}_{j=1}^n}\varDelta Qj} $$

where:

*Q*: Amount (%) of the drug released at the time (*t*)
*Q*max: Maximum amount of the drug released (=100%)
*n*: Number of timepoints
Δ*Q*: *Q*_t_–*Q*_t-1_
*t*^AV^_*j*_: (*t*_i_ + *t*_i-1_)/2

### Transmittance Analysis

The samples of SE powder were analyzed for transmittance using the UV–VIS spectrophotometer Jasco VT-530 (Japan) at 520 nm wavelength. Prior to the measurement, the samples corresponding to 50 mg of ibuprofen were dispersed in 900 mL of distilled water under continuous stirring at 37°C and 50 rpm. The transmittance was measured in triplicate after 60 min. The one-way ANOVA test was applied to evaluate the differences between transmittance values for different formulations. The significance was assumed, if *P* value was below 0.05.

## RESULTS AND DISCUSSION

Taking into account the advantages of solid dosage forms in comparison to liquids as well as a beneficial effect of mesoporous silicates on ibuprofen dissolution and stability, the opportunity to convert ibuprofen solution in Labrasol into SE powder, using either the alkaline or neutral grade of magnesium aluminometasilicate, is examined in the present study.

Magnesium aluminometasilicates are excipients certified to be used in pharmaceutics, which are manufactured on an industrial scale (16). Many of other porous carriers of well-defined pore size and much higher specific surface area, *i.e.*, SBA-15 or MCM-41 are synthetized at the laboratory scale so as it may be difficult to use them in commercial technologies. Furthermore, their application for the drug delivery at an industrial scale may be limited, especially if a multistage organic solvent impregnation is necessary. Therefore, detailed characteristics of commercially available mesoporous amorphous carriers as well as a single-stage impregnation technology can be helpful during the development of either innovative or generic drugs.

### Sample Characterization

The results presented in Table I show that the consistency of samples changes from liquid to semi-solid and finally solid with decreasing Labrasol content and increasing the silicate amount.

Among eighteen formulations prepared, thirteen are in the powder form (Table I). Two formulations, S2 and U2, are semi-solid, and three formulations, S3, S6, and U3, are liquid. An increase in Labrasol amount from 150 to 300–600 μL results in the formation of agglomerates.

In general, a higher amount of liquid formulation is adsorbed if Neusilin US2 is used as a solid carrier in comparison to Neusilin SG2. Seven out of nine formulations U are solid (Table I). The application of Neusilin SG2 enables to obtain only three S-SE formulations without agglomerates, *i.e.*, S4, S7, and S8. The presence of agglomerates is visible in three other solid samples such as S1, S5, and S9.

Taking into account the amount of the solid carrier, it has been shown that all the formulations containing 300 mg of the silicate are solid. If 200 or 100 mg of the silicate is used, the consistency of the sample depends on the kind of the silicate.

The differences in consistency can result from the physicochemical properties of Neusilin US2 and SG2. Agarwal *et al.* (7) report that liquid SE formulation is usually adsorbed on the surface of a solid carrier but it is also filled in its pores. Therefore, the effectiveness of the adsorption process depends on the particle size of the carrier and its specific surface area.

**Fig. 1:**
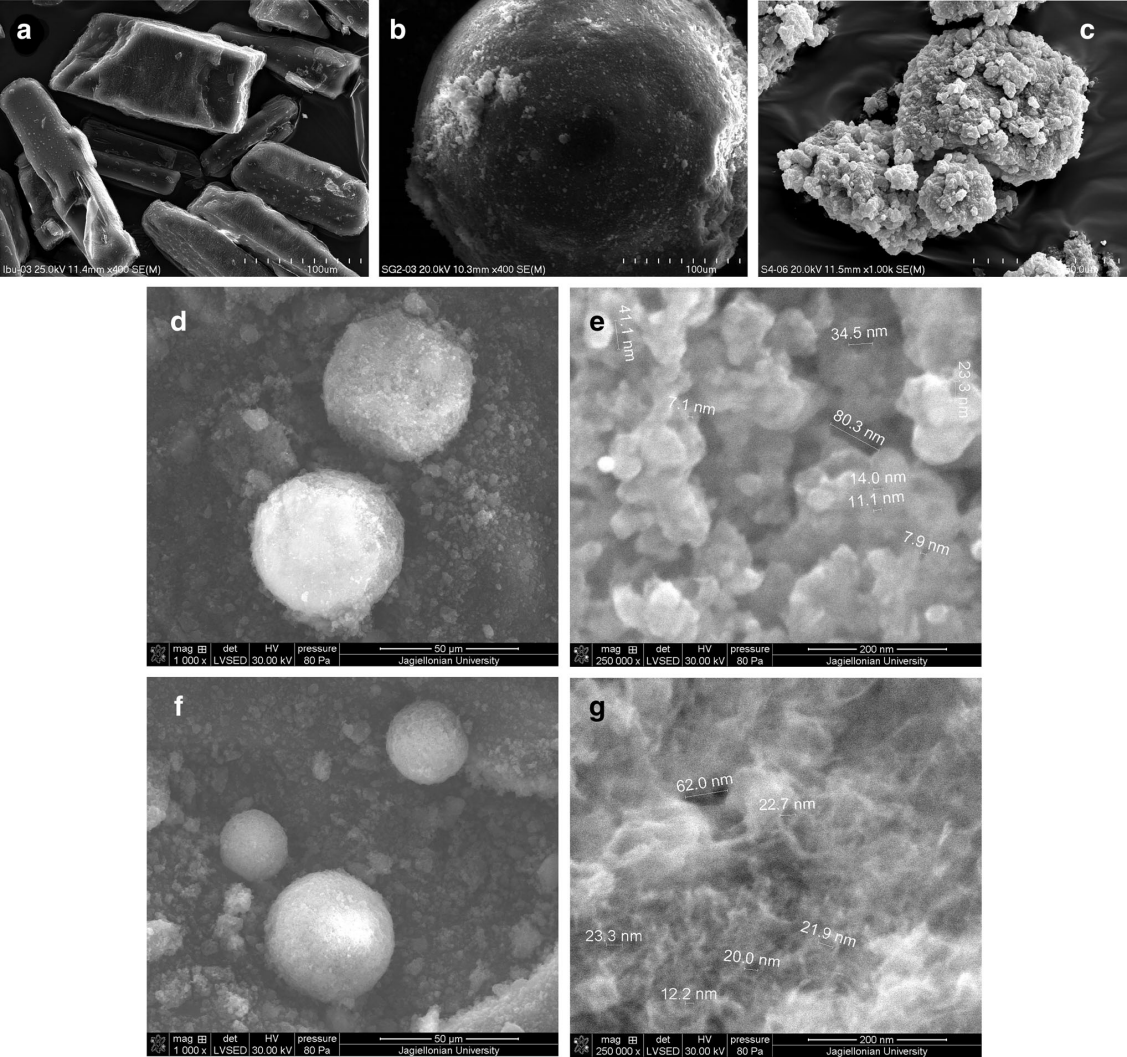
SEM pictures of ibuprofen (**a**), Neusilin SG2 (**b**), and S4 (**c**). Low vacuum SEM pictures of Neusilin US2 (**d, e**) and U7 (**f, g**)

Two grades of spray-dried magnesium aluminometasilicates investigated in the present study are spherical granules that differ in particle size (Fig. 1). Particles of Neusilin US2 are about three times smaller than Neusilin SG2.

The porosity of both grades of magnesium aluminometasilicate was examined by the nitrogen adsorption method (Table II). The nitrogen adsorption and desorption isotherms of the silicates form a distinct hysteresis loop, which confirms the presence of mesopores in the samples (data not shown here). According to IUPAC nomenclature, the diameter of mesopores ranges from 1.7 to 50 nm.

**Table II:** Texture of Magnesium Aluminometasilicates and Selected Solid SE Formulations Determined by Nitrogen Adsorption Method

Parameters	Neusilin SG2	S4	Neusilin US2	U7
Amount of IBU per 1 g of sample (wt%)	–	20	–	14
Amount of silicate per 1 g of sample (wt%)	100	80	100	86
Specific surface area, S_BET_ (m^2^/g)	186.51	0.52	371.88	36.87
Micropore area, S_micro_ (m^2^/g)	24.42	0.30	63.64	0
Mesopore volume, V_mezo_^a^ (cm^3^/g)	0.6792	0.0002	1.5379	0.3247
Micropore volume, V_micro_ (cm^3^/g)	0.0096	0.00006	0.0267	0
Mesopore diameter, D_mezo_^b^ (nm)	12.85	18.47	14.47	23.63

^*a*^BJH desorption cumulative pore volume in the range from 1.7 to 300 nm

^*b*^BJH desorption cumulative pore diameter in the range from 1.7 to 300 nm

The texture parameters calculated on the basis of nitrogen isotherms are provided in Table II. The specific surface area of Neusilin US2 is twice as high as in Neusilin SG2, so is the mesopore volume, which is more than twice as high (Table II). The average mesopore diameter ranges from 12.85 to 14.47 nm, and Neusilin US2 has slightly bigger pores than the alkaline grade. There is also a small volume of micropores, *i.e.*, pores <1.7 nm in diameter, in both excipients. The micropore area of Neusilin US2 is 2.6-fold as high as in Neusilin SG2.

The diffractograms of SE powders and their solid components confirm the crystallinity of ibuprofen and the amorphous form of both magnesium aluminometasilicates (data not shown here). After the adsorption of drug solution, all the samples seem to be amorphous. The Bragg’s peaks corresponding to crystalline ibuprofen in the range from 6 to 40° at 2Θ are not found in solidified SE samples. Similar results are obtained for the physical mixtures. Crystalline ibuprofen dry-blended with silicates is converted into the amorphous form.

The percentage of *W*_max_ determines an amount of water incorporated into the formulation, which causes a permanent turbidity of the system (26). In the present study, distilled water was being gradually added to 0.3 or 0.6 mL of the surfactant. The mixture became turbid but turbidity disappeared during gentle stirring. The calculated values of *W*_max_ values for Labrasol ranged from 98.7 to 98.9% for 0.6 or 0.3 mL of the surfactant. The transmittance of samples was 16 and 18%, respectively. Dilution of the samples with water resulted in an increase in their clarity. The transmittance of the sample containing 0.3 mL of Labrasol and 900 mL of water was 97%, whereas in the case of the mixture containing twice as high as an amount of Labrasol, the transmittance was 90%.

The clarity of S-SE formulations after their dispersion in water was analyzed by transmittance measurements. The results show that for all the formulations investigated, transmittance ranges from 78 to 100%. The transmittance of *placebo* formulations S1, S4-5, U1, U4-6, and U9 is similar to those containing ibuprofen (*P* < 0.05). Furthermore, the kind of the carrier has no influence on the transmittance of SE powder dispersed in water. An increase in the silicate content to 300 mg or/and adsorption of 600 μL of the liquid formulation on its surface causes a decrease in the formulation clarity. Formulations S9, U9, and U6 have the lowest transmittance, ranging from 78 to 82%. The formulations containing the lowest number of excipients have the highest transmittance of about 100% and the best clarity.

### Drug Release

It has been reported that the dissolution rate of the drug from S-SE increases with the decreasing particle size of the solid carrier (11). Small particles of a high specific surface area have higher contact with SE formulation as compared to big particles. Ito *et al.* (9) described the properties of S-SE formulations prepared by the adsorption of gentamycin sulfate dispersed in Labrasol on the surface of silicates, where the specific surface area varied from 120 to 300 m^2^/g. SE powder was then placed into hard gelatin capsules. The results of both dissolution and bioavailability studies showed that the highest amount of gentamycin was dissolved from the samples containing the silicate of the lowest specific surface area, *i.e.*, 120 m^2^/g. They concluded that the carriers with a low specific surface area could be more effective in dissolution rate improvement as compared to the carriers of a high specific surface area (9). Due to the fact that the porous carriers of a high specific surface area may be less dispersed in the gastrointestinal tract, API dissolution and adsorption can be slower. Furthermore, if strong adsorbents are used to solidify APIs solutions in Labrasol, the drug release rate can be sustained (9).

In the present study, dissolution tests were carried out, using two different solvents, such as HCl 0.1 mol/L without pepsin of pH = 1.2 and the pharmacopoeial phosphate buffer of pH = 7.2 recommended for ibuprofen. Since the solubility of ibuprofen in pH = 1.2 is very low, *i.e.*, 0.038 mg/mL, drug dissolution studies in 0.1 mol/L HCl were performed with the aim to determine if the presence of a self-emulsifying surfactant and a silicate solid carrier could improve its dissolution.

**Fig. 2:**
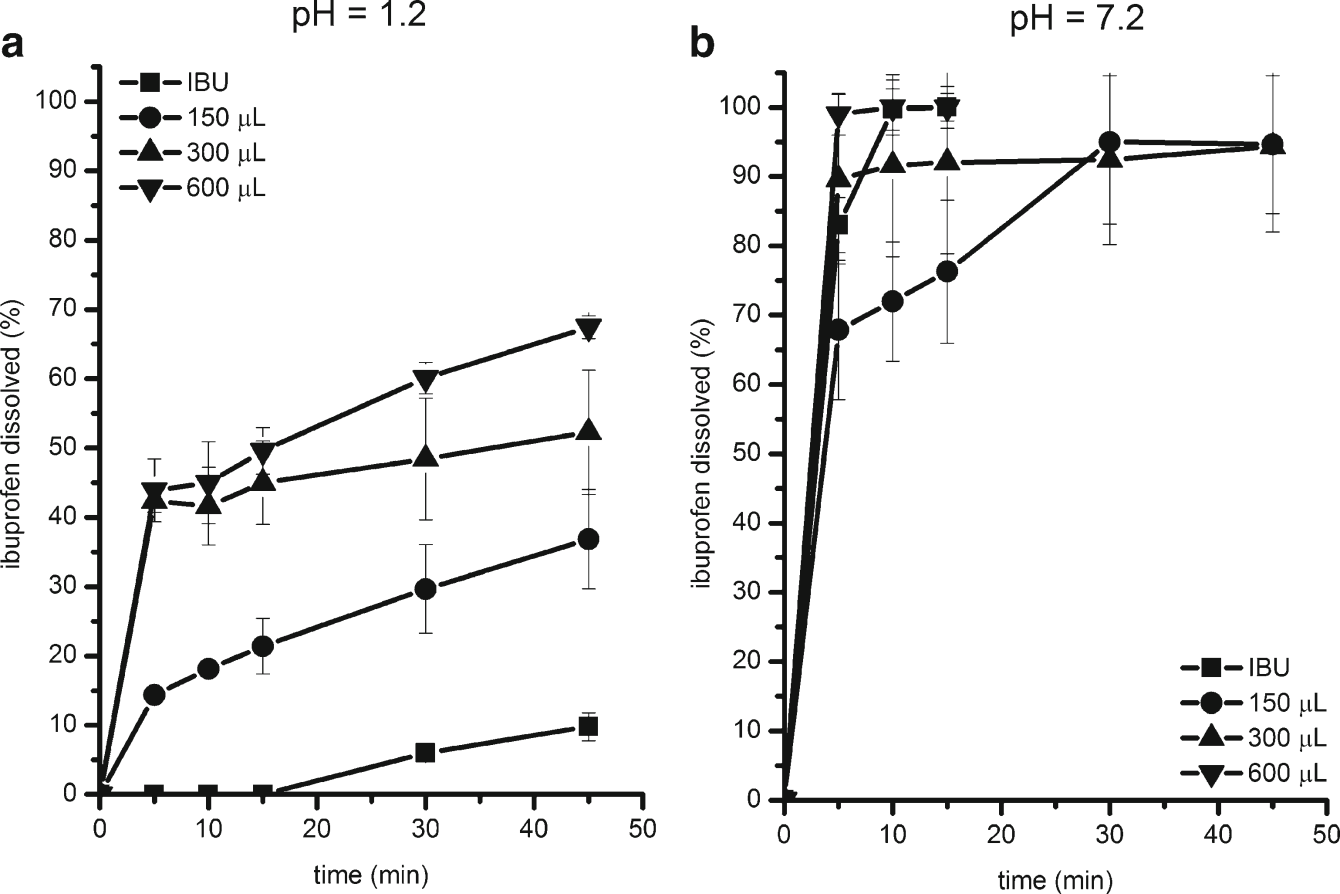
Influence of Labrasol content on ibuprofen dissolution from liquid SE formulations composed of 50 mg of ibuprofen and 150–600 μL of Labrasol: HCl 0.1 mol/L of pH = 1.2 (**a**), phosphate buffer of pH = 7.2 (**b**). *Error bars* represent SD of three measurements

The dissolution curves of liquid self-emulsifying formulations are presented in Fig. 2. After 45 min, no more than 67% of ibuprofen is released in pH = 1.2 (Fig. 2a). In spite of the fact that the application of Labrasol enables to improve significantly (*P* < 0.05) the amount of ibuprofen dissolved, the dissolution improvement was the highest at the beginning of the test. After 5 min, the amount of ibuprofen released was from 15 to 45 times as high as in the pure drug. Finally, after 45 min, the amount of the drug dissolved increased from about four to seven times with the increasing amount of Labrasol in the formulation. Fine droplets of SE lipid formulation have been visible until the end of the dissolution test, which can confirm that not all ibuprofen was dissolved after 45 min.

The dissolution profiles in the phosphate buffer show that after 30 min, more than 80% of ibuprofen is dissolved from all the liquid lipid formulations (Fig. 2b). This fact may indicate an immediate drug release.

**Fig. 3:**
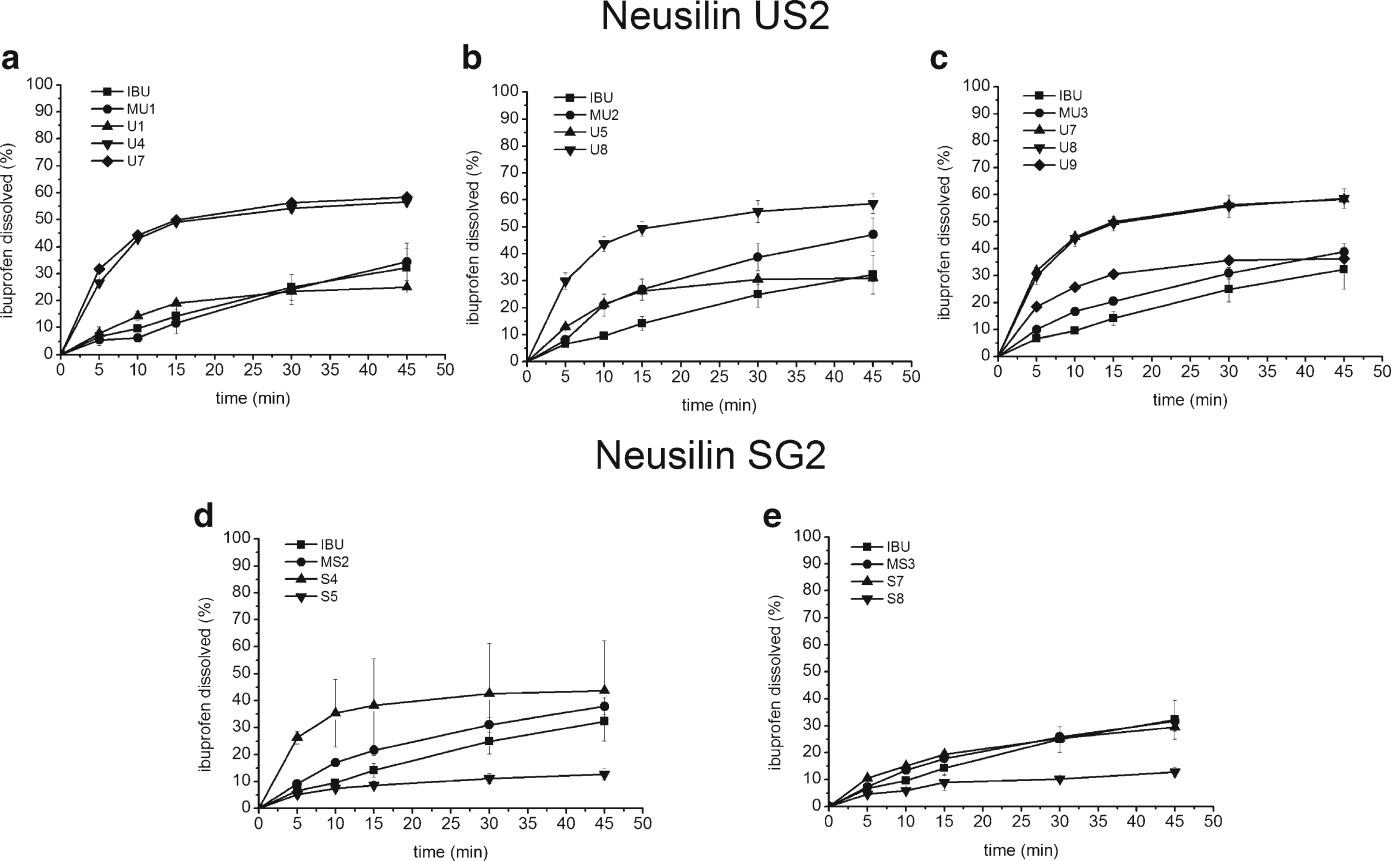
Dissolution profiles of S-SE formulations containing Neusilin SG2 (**a**, **b**, **c**) and Neusilin US2 (**d**, **e**) as solid carrier in pH = 1.2. *Error bars* represent SD of three measurements

The dissolution curves of S-SE formulations and physical mixtures in HCl 0.1 mol/L are shown in Fig. 3. The results confirm that the kind of the silicate and the content of Labrasol in the S-SE formulation have influence on the ibuprofen release rate. After 45 min, the highest amount of ibuprofen, *i.e.*, more than 50%, dissolved from samples U7 and U8, containing Neusilin US2 (Fig. 3a–c). The amount of ibuprofen dissolved from formulations S was lower (Fig. 3d, e). In such a case, the best properties were observed in formulation S4, from which more than 40% of the drug was dissolved in pH = 1.2 after 45 min (Fig. 3d).

The model-independent parameters such as mean dissolution time (MDT) and dissolution efficiency (DE), calculated on the basis of ibuprofen dissolution profiles in HCl 0.1 mol/L, show that formulation S4 has the shortest MDT such as 7 min and the highest DE of 37% (Table III). The comparison of the model-independent parameters between S4 and S7 reveals that an increase in the silicate content from 200 to 300 mg causes an approximately twofold increase in MDT, *i.e.*, from 7 to 14 min and an 18-fold decrease in ibuprofen DE which is only of 2%. If the content of the silicate is the same (S7 and S8), but if there is twice more liquid SE formulation adsorbed (S8), DE can be higher such as 9%.

**Table III:** Model-Independent and Model-Dependent Parameters for S-SE Formulations as Well as *P* Values of ANOVA Test

Sample name		MS1	MS2	MS3	S4	S7	S8	MU1	MU2	MU3	U1	U4	U5	U7	U8	U9
MDT [min]		21.44	16.05	16.40	7.05	13.72	14.46	21.91	16.37	16.83	11.40	8.33	8.75	7.97	8.58	8.33
DE [%]		14.31	24.38	20.16	36.85	2.05	8.68	17.69	29.96	24.30	18.61	46.14	25.00	48.05	47.50	29.64
Zero order	K r^2^	0.5814 0.9920	0.6778 0.9548	0.5797 0.9584	0.3669 0.8000	0.4516 0.9472	0.1935 0.9170	0.7654 0.9906	0.8954 0.9252	0.6954 0.9790	0.3852 0.8140	0.6119 0.6939	0.3939 0.7317	0.5716 0.7667	0.6116 0.7693	0.4044 0.7693
Korsmeyer-Peppas model	K n r^2^	1.2001 0.802 0.9632	3.6584 0.630 0.9798	2.7910 0.654 0.9829	19.8573 0.222 0.8784	5.1729 0.466 0.9920	2.1756 0.468 0.9551	1.0357 0.906 0.9459	2.9350 0.763 0.9320	3.8894 0.610 0.9955	3.9531 0.517 0.9133	18.1398 0.323 0.8494	7.9108 0.391 0.8808	22.4343 0.268 0.9140	20.5224 0.294 0.9062	12.1172 0.309 0.9324
Hixson-Crowell model	K r^2^	0.0334 0.9853	0.0284 0.8806	0.0277 0.8858	0.0113 0.7725	0.0207 0.8979	0.0160 0.8712	0.0394 0.9678	0.0343 0.8107	0.0286 0.9259	0.0199 0.7366	0.0168 0.6366	0.0165 0.6734	0.0148 0.7207	0.0161 0.7159	0.0146 0.7461
*P* values		n.s.	<0.001	<0.001	n.s.	<0.01	n.s.	n.s.	<0.01	<0.001	<0.01	<0.001	<0.01	<0.001	<0.001	<0.001

*n.s*. non significant, mean dissolution time (MDT), dissolution efficiency (DE)

DE and MDT values for solid self-emulsifying samples containing Neusilin US2 depend also on the silicate content and the amount of liquid formulation incorporated into the solid carrier (Table III). The analysis of the formulation U4 and U5 shows that a twofold increase in the amount of Labrasol causes an almost twofold decrease in DE but MDT is similar. However, the analysis of SE powders U7 and U8 shows similar results to U4, *i.e.*, MDT of about 8 min and DE of about 48%. If there is 300 mg of the silicate, an increase in Labrasol content to 600 μL causes a decrease in DE from 48 to 30%, but MDT is still about 8 min.

The dissolution of S-SE consisting of Neusilin US2, examined in HCl 0.1 mol/L, shows better results as compared to the physical mixtures (Fig. 3; Table III). For the majority of U samples, MDT is shorter and the dissolution efficiency is higher than for MU. The results for the physical mixtures composed of ibuprofen and Neusilin SG2 indicate that except for S4, DE is lower than S-SE (Table III).

**Fig. 4:**
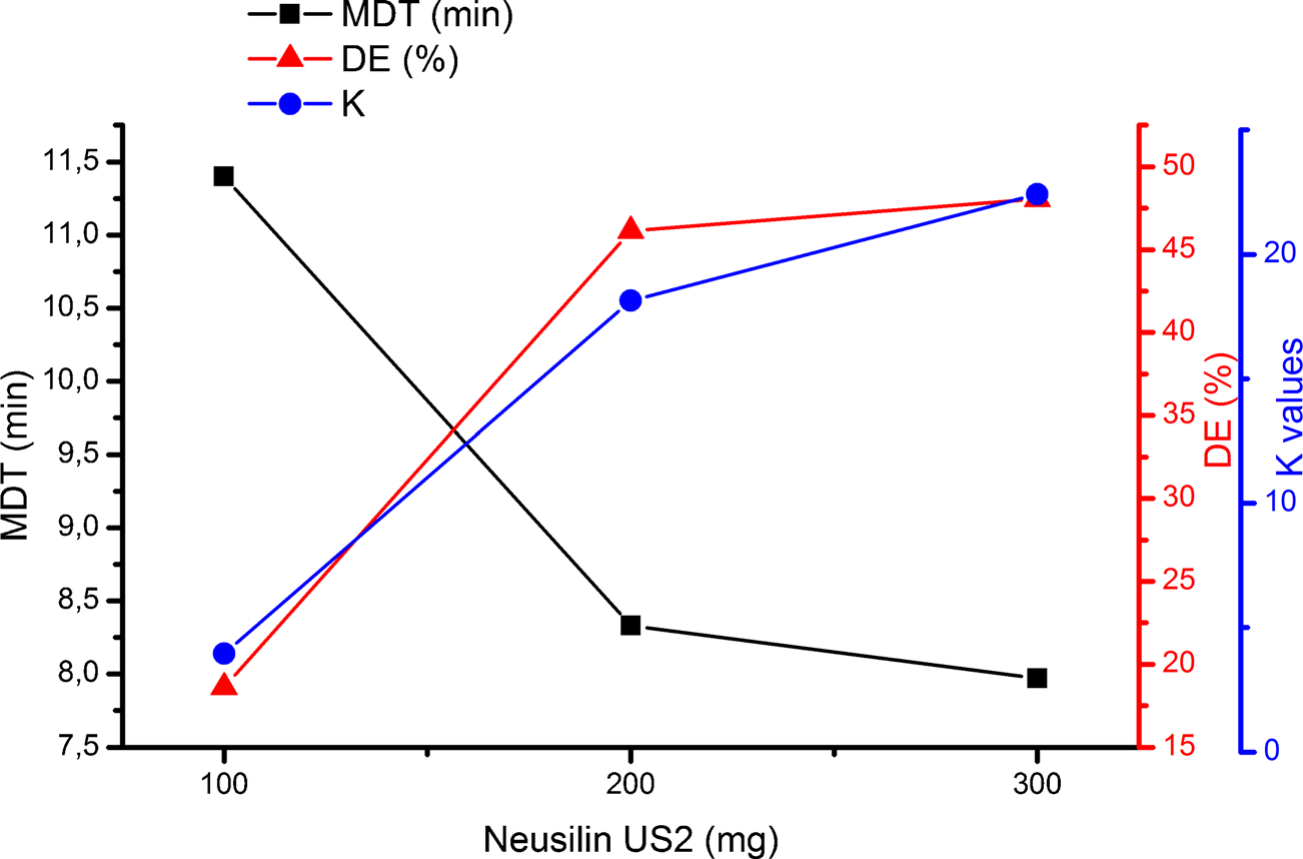
Influence of amount of Neusilin US2 on ibuprofen dissolution rate (*K*), MDT, and DE from S-SEDDS

Furthermore, the comparison of dissolution profiles for formulations U1, U4, and U7 containing 100, 200, and 300 mg of Neusilin US2 reveals that MDT decreases but the dissolution rate based on diffusion (K) and DE increases with increasing the silicate content (Fig. 4).

In order to explain the drug dissolution mechanism, the dissolution profiles were fitted to the zero order, first order, Korsmeyer-Peppas, Michaelis-Menten, Higuchi, and Hixson-Crowell mathematical models (24,25). The highest values of coefficients of determination (*r* ^2^) were found for three of these models, *i.e.*, the zero order, Korsmeyer-Peppas, and Hixson-Crowell. The one-way ANOVA test was performed for each dissolution profile to evaluate if the values of *r* ^2^ calculated for the three models are significantly different (Table III). The results for S-SE containing Neusilin SG2 as a solid carrier indicate that only in the case of formulation S7, the differences in *r* ^2^ values are significant (*P* < 0.05). In such a case, the diffusion-based mechanism of the drug release may be suggested.

Different results can be seen from Table III for Neusilin US2. The coefficients of determination are significantly different (*P* < 0.05) for all S-SE formulations. The highest values of *r* ^2^ may suggest that ibuprofen can be dissolved by diffusion (*r* ^2^ 0.8494–0.9342) rather than by the erosion-based mechanism (*r* ^2^ 0.6366–0.7461) or the zero order kinetics (*r* ^2^ 0.6939–0.8140).

The goodness of fit was also evaluated using chi-square (*χ* ^2^) test and Akaike information criteria (AIC). The results are shown in Table IV. For at least one model, seven formulations, *i.e.*, MS1, MS2, S4, S7, S8, MU3, and U9 have lower chi-square values (*χ* ^2^) than the critical value. For a given formulation, the smallest AIC was used to select the most appropriate model, giving the best fit. Korsmeyer-Peppas model fits the best if Neusilin SG2 is used as a carrier, which confirms that ibuprofen can dissolve by diffusion. However, the mechanism of ibuprofen release from physical mixtures containing Neusilin SG2 is of a mixed nature and it may be either zero order or diffusion based. Chi-square values for formulations containing Neusilin US2 such as MU3 and U9 are lower than 0.7102 (Table IV). There are no statistically significant differences between Korsmeyer-Peppas mathematical model and the data points. Therefore, regardless the statistical test used to evaluate the goodness of fit, it is difficult to draw a general conclusion with regard to ibuprofen dissolution mechanism.

**Table IV:** Goodness of Fit Evaluation Results (*χ* ^2^, *P* value) for S-SE Formulations, Containing Neusilin SG2 and US2

Formulation name	Zero order model	Korsmeyer-Peppas model	Hixson-Crowell model						
	Chi-square (*χ* ^2^)	*P* value	AIC	Chi-square (*χ* ^2^)	*P* value	AIC	Chi-square (*χ* ^2^)	*P* value	AIC
MS1^a^	**0.3701**	**0.9848**	**9.3379**	0.6962	0.9518	15.1140	0.4494	0.9782	14.9412
MS2^a^	1.4042	0.8435	19.7460	**0.3979**	**0.9826**	**12.4787**	2.5938	0.6279	23.8383
MS3	6.0720	0.1938	17.6765	4.7267	0.3165	10.4169	8.0230	0.0907	22.1093
S4^a^	1.4469	0.8360	23.5059	**0.5325**	**0.9703**	**18.4196**	1.5980	0.8091	24.0722
S7^a^	0.7277	0.9479	16.4913	**0.1042**	**0.9987**	**6.7114**	1.1900	0.8797	19.4587
S8^a^	0.5251	0.9710	10.4454	**0.2569**	**0.9924**	**7.3447**	0.7267	0.9480	12.2412
MU1	4.0774	0.8501	12.8986	4.8705	0.7713	16.3994	5.9726	0.6503	19.8749
MU2	17.5402	0.1646	25.1937	8.7402	0.3647	21.1080	19.8549	0.0109	28.9917
MU3^a^	2.1128	0.9774	16.0404	**0.3749**	**0.99996**	**4.0270**	4.8600	0.7724	21.5308
U1	2.4624	0.6514	21.9602	1.1525	0.8859	17.9180	3.2355	0.5192	23.6819
U4	4.4397	0.3498	29.8802	2.1847	0.7018	26.1641	4.8154	0.2841	30.6033
U5	2.8595	0.5816	24.5784	1.3473	0.8533	20.6985	3.0608	0.4940	25.6116
U7	2.4575	0.6523	27.3513	0.8969	0.9250	22.2153	2.5769	0.5930	28.0965
U8	2.8666	0.5804	27.9286	1.1200	0.8911	23.0590	3.0936	0.5111	28.7315
U9^a^	1.7307	0.7851	23.2262	**0.5975**	**0.9633**	**17.9756**	1.7627	0.7276	24.2043

Chi-square critical value for α = 0.05 (lower-tail) and degrees of freedom (df) equal to 4 is 0.7102

*χ* ^*2*^ chi-square, *P Value* probability of null hypothesis acceptance, *AIC* Akaike Information Criterion

^*a*^discussed formulations, bolded values of models giving best fit

Since ibuprofen is an organic acid and its dissolution depends on pH, it seems that the application of an alkaline solid carrier, *i.e.*, Neusilin SG2, may be helpful to improve its dissolution in low pH. Nevertheless, the results show that the amount of ibuprofen dissolved from formulations S, prepared using the alkaline solid carrier, is lower than from U samples, containing neutral grade of magnesium aluminometasilicate. These results are in agreement with the literature reports (5,6,16,27). The loading of acidic drugs into amino-functionalized mesoporous silica can be applied to suppress the dissolution rate. This effect is due to strong interactions between the amine and carboxylic group. Rosenholm and Linden (27) claims that the result is pronounced if there is a very high excess of amino functions on the silicate surface. According to the data provided by the manufacturer, pH of 4% aqueous slurry prepared using Neusilin SG2 is from 8.5 to 10.0.

In the present study, a small amount of ibuprofen dissolved from formulations S can also be related to the differences in the texture of mesoporous carriers. SEM pictures of solid self-emulsifying formulations S4 and U7 are presented in Fig. 1. Particles of U7 are spherical, whereas the shape of S4 particles is irregular. The image analysis performed using the high resolution scanning electron microscopy shows the presence of mesopores on the carrier surface. After impregnation with liquid SE, the surface of the particles becomes smooth, which can be due to the fact that the pores are filled. These findings are in agreement with the results of texture analysis obtained by nitrogen adsorption method.

**Fig. 5:**
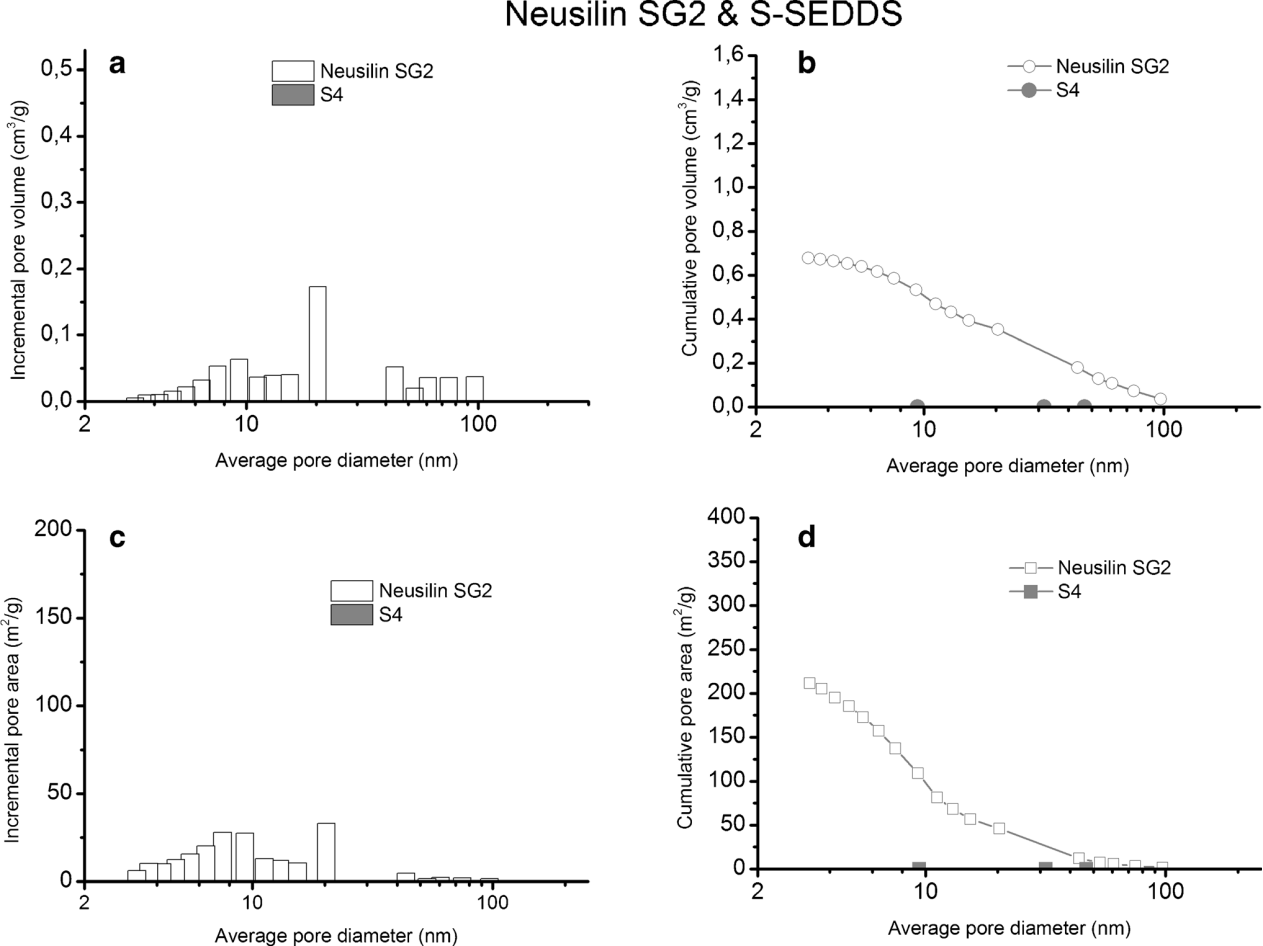
Pore size distribution (**a**), cumulative pore volume (**b**), pore area distribution (**c**), and cumulative pore area (**d**) in Neusilin SG2 (*white bars* and *symbols*) and S4 (*grey bars* and *symbols*)

**Fig. 6:**
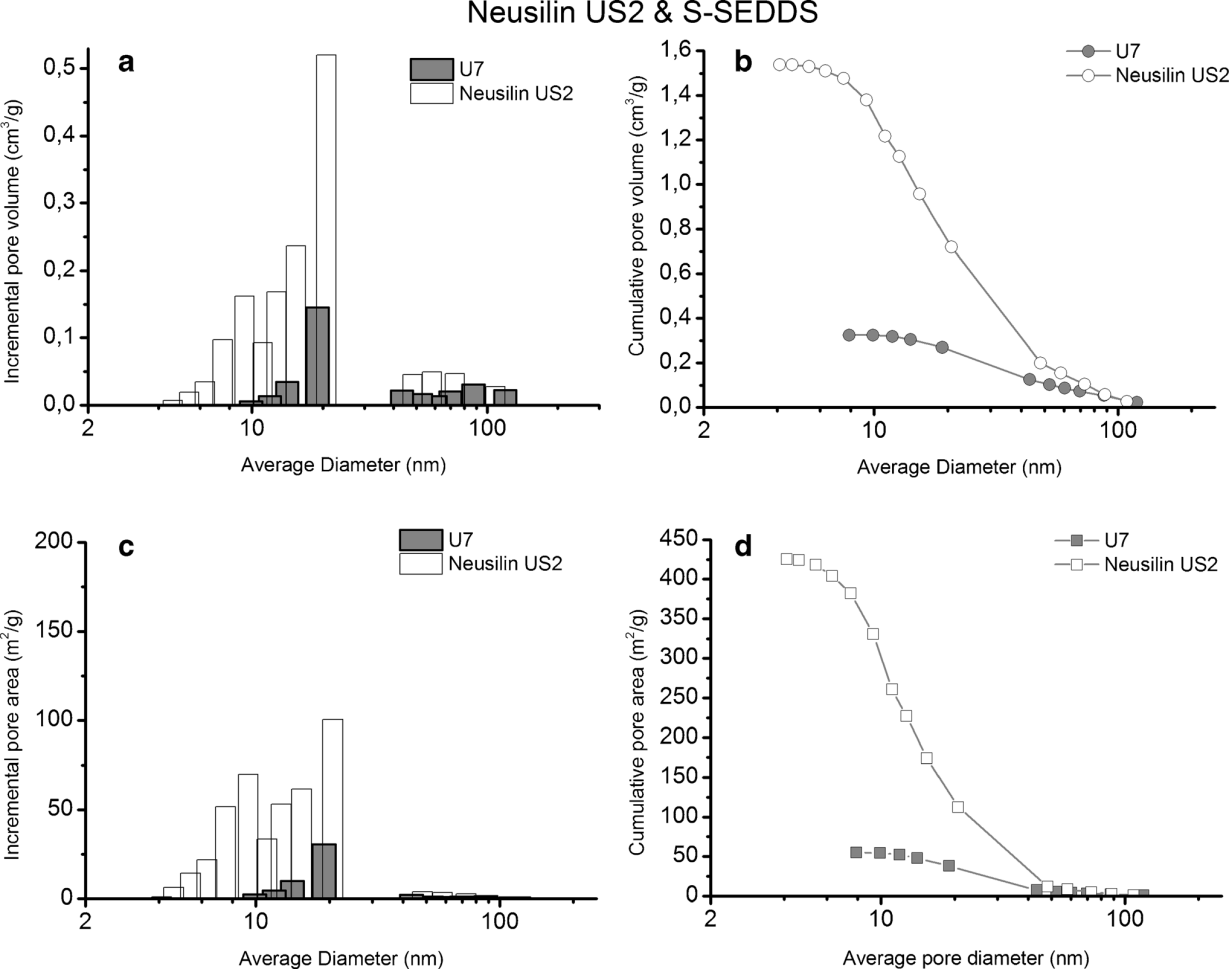
Pore size distribution (**a**), cumulative pore volume (**b**), pore area distribution (**c**), and cumulative pore area (**d**) in Neusilin US2 (*white bars* and *symbols*) and U7 (*grey bars* and *symbols*)

The pore size distribution and the pore area of pure magnesium aluminometasilicates and solid self-emulsifying formulations S4 and U7 are shown in Figs. 5 and 6. It can be seen from Figs. 5a and 6a that the pores of 20.3 nm in diameter dominates in both Neusilin SG2 and Neusilin US2. Apart from that, there are also pores in the range of 4–15 nm and 40–100 nm but the pore volume and the pore area of Neusilin SG2 are more than twice as low as in Neusilin US2 (Figs. 5–6b–d, Table II).

In the case of S4, the adsorption of 150 μL of the liquid lipid formulation on 200 mg of Neusilin SG2 causes a complete filling of pores (Fig. 5). The specific surface area, S_BET_, for sample S4 is 0.52 m^2^/g, whereas S_BET_ for the pure carrier is 186.51 m^2^/g (Table II). The texture analysis of formulation U7, consisting of Neusilin US2, confirms that the adsorption of the same amount of liquid formulation on 300 mg of the silicate results also in a decrease in the pore volume as well as the pore area. S_BET_ of formulation U7 decreases more than ten times in comparison to the pure carrier (Table II). Figure 6 confirms that the pores below 10 nm and a half of the pores from 40 to 100 nm in Neusilin US2 are filled with the liquid lipid formulation.

**Fig. 7:**
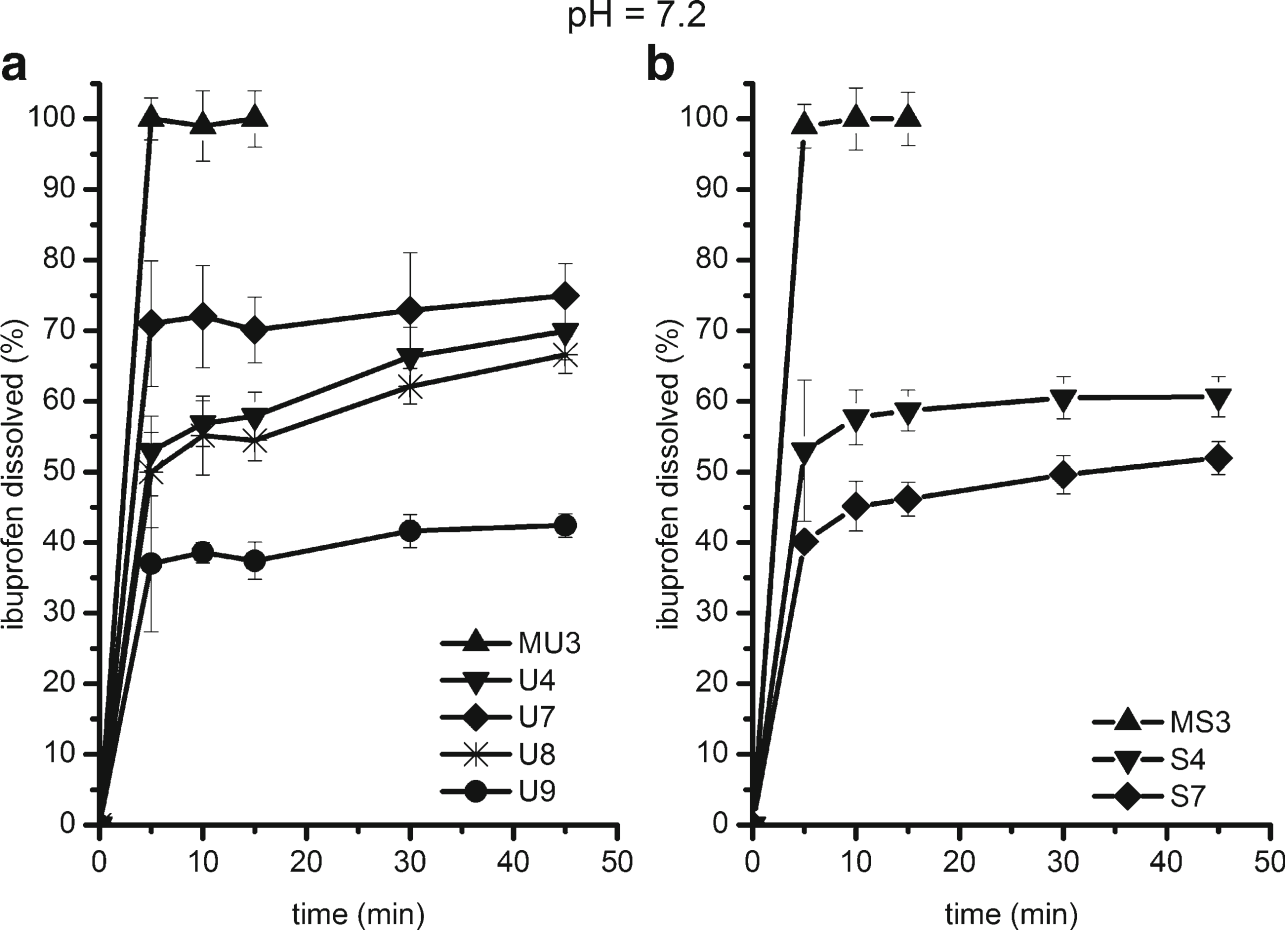
Dissolution profiles of ibuprofen from S-SEDDS consisting of Neusilin US2 (**a**) and Neusilin SG2 (**b**) in pH = 7.2. *Error bars* represent SD of three measurements

Figure 7 shows the dissolution profiles of the selected solid self-emulsifying formulations in phosphate buffer of pH = 7.2. The release of ibuprofen from the physical mixtures MU3 and MS3 as well as the dissolution of the pure drug is immediate (more than 80% in 5 min). In spite of this, the sustained release of ibuprofen was obtained from all S-SE. Among the formulations consisting of Neusilin US2, the highest amount of ibuprofen, *i.e.*, more than 70%, was dissolved after 45 min from U7. The higher amount of the liquid lipid formulation adsorbed on the solid carrier caused a decrease in the amount of ibuprofen dissolved to 60 or 40% for U8 or U9, respectively (Fig. 7a). However, the dissolution profiles of U4 and U7 indicate that an increase in the carrier content can improve ibuprofen dissolution if the amount of the liquid SE formulation is the same.

The amount of ibuprofen dissolved after 45 min from S4 and S7, containing Neusilin SG2, was lower, *i.e.*, 50 and 40%, respectively. In this case, an increase in the silicate content caused a decrease in the drug release. It might be related to strong interactions between the acidic drug and the alkaline silicate or to the insufficient porosity of the carrier for the effective wetting of the drug entrapped in its pores (Fig. 7b).

Therefore, the application of the neutral or alkaline mesoporous magnesium aluminometasilicates as carriers for ibuprofen solutions in Labrasol may give the opportunity to obtain the modified drug release. The advantage of such formulations is the lack of the residual organic solvents and the minimal number of the excipients used for their preparation (5,6,14,20,28).

## CONCLUSIONS

The application of magnesium aluminometasilicate enables to prepare S-SEDDS with amorphous ibuprofen, using Labrasol as a lipid self-emulsifying excipient by simple blending of components. The application of an additional organic solvent is not necessary to impregnate the solid carrier with the liquid formulation, which is an important advantage.

The liquid lipid formulation is adsorbed in the pores of silicates. The adsorption of the lowest amount of the liquid SE causes the filling of all the pores in Neusilin SG2. It results in the decrease in ibuprofen dissolution as the wetting of the drug entrapped in the pores is more difficult. Strong interactions between the carboxylic group of the drug and the surface of the alkaline silicate may also sustain the release of acidic drugs. Thus, the application of the neutral silicate can be more effective for the dissolution rate improvement of ibuprofen.

The dissolution efficiency is the highest, *i.e.*, 37–48% and mean dissolution time is the shortest, *i.e.*, about 8 min for samples S4 and U7. The amount of Labrasol in the formulation, the physicochemical properties of both the drug, and the solid carrier are critical parameters to optimize ibuprofen release.
